# High-Performance Biobased Unsaturated Polyester Nanocomposites with Very Low Loadings of Graphene †

**DOI:** 10.3390/polym10111288

**Published:** 2018-11-20

**Authors:** Chengguo Liu, Cuina Wang, Jijun Tang, Jing Zhang, Qianqian Shang, Yun Hu, Hongxiao Wang, Qiong Wu, Yonghong Zhou, Wen Lei, Zengshe Liu

**Affiliations:** 1 Institute of Chemical Industry of Forest Products, Chinese Academy of Forestry, Key Lab of Biomass Energy and Material, Jiangsu Province, National Engineering Lab for Biomass Chemical Utilization, Key Lab on Forest Chemical Engineering, State Forestry Administration, Nanjing 210042, China; liuchengguo@icifp.cn (C.L.); Shang_qianqian@126.com (Q.S.); 15150509893@139.com (Y.H.); wanghongxiao_1@163.com (H.W.); 2 College of Science, Nanjing Forestry University, Nanjing 210037, China; wangcuina66@163.com (C.W.); wqzjj9394@163.com (Q.W.); 3 Department of Corrosion Prevention and Polymer Materials, College of Materials Science and Engineering, Jiangsu University of Science and Technology, Zhenjiang 212003, China; tangjijunnju@126.com (J.T.); badbush2003@163.com (J.Z.); 4 Bio-Oils Research, National Center for Agricultural Utilization Research, Agricultural Research Service, United States Department of Agriculture, 1815 N. University St., Peoria, IL 61604, USA

**Keywords:** graphene, unsaturated polyester resins, tung oil, biobased polymer nanocomposites, in situ melt polycondensation

## Abstract

Graphene-reinforced tung oil (TO)-based unsaturated polyester nanocomposites were prepared via in situ melt polycondensation intergrated with Diels–Alder addition. Functionalized graphene sheets derived from graphene oxide (GO) were then extracted from the obtained nanocomposites and carefully characterized. Furthermore, dispersion state of the graphene nanosheets in the cured polymer composites and ultimate properties of the resultant biobased nanocomposites were investigated. Mechanical and thermal properties of the TO-based unsaturated polyester resin (UPR) were greatly improved by the incorporation of GO. For example, at the optimal GO content (only 0.10 wt %), the obtained biobased nanocomposite showed tensile strength and modulus of 43.2 MPa and 2.62 GPa, and *T*_g_ of 105.2 °C, which were 159%, 191%, and 49.4% higher than those of the unreinforced UPR/TO resin, respectively. Compared to neat UPR, the biobased UPR nanocomposite with 0.1 wt % of GO even demonstrated superior comprehensive properties (comparable stiffness and *T*_g_, while better toughness and thermal stability). Therefore, the developed biobased UPR nanocomposites are very promising to be applied in structural plastics.

## 1. Introduction

Unsaturated polyester resins (UPRs) are widely utilized in industrial and domestic areas due to their low cost, ease of handling, and good balance of mechanical, thermal, electrical, and chemical resistant properties [1,2]. However, with the current concerns on exploring alternatives to petroleum and reducing environmental pollution, research is being increasingly directed to develop polymeric materials from renewable resources such as proteins, oils, and carbohydrates [3,4,5]. Among all the biomass-derived feedstocks, plant oils are the primary choice to prepare UPRs because of their abundance, low toxicity, biodegradability, and triglyceride structures suitable for further chemical modification [6,7]. As a result, plant oil-based UPRs have attracted considerable attention since 2000.

Blending plant oils or their derivatives with petroleum-based UPRs is an efficient strategy to to prepare oil-based UPRs [8,9,10,11,12,13,14]. The addition of flexible oil-based modifiers usually leads to an apparent improvement of toughness compared to petroleum-based UPRs, however, a large loss of stiffness is commonly observed in the resulting biobased UPRs when the content of oil-based modifiers are not so high (10–20 wt %), thus leading to the unbalance of stiffness and toughness for the biobased UPRs. A good solution to address the obstacle is to reinforce the bioresins with nanofillers [11,12,13,15,16]. Biobased UPRs enhanced with a small amount of nanoclays were shown to exhibit good enhancements in mechanical, thermal, and barrier properties, whereas stiffness and other properties were only partially recovered [12,13]. Hence, exploring other nanofillers which are more efficient in the improvement of stiffness is an important task for the real application of such biobased UPRs.

Graphene, a single-atom-thick 2D sheet of sp^2^-hybridized carbon atoms, has been extensively utilized in polymer nanocomposites owing to its superior properties like high mechanical stiffness (~1.0 TPa) and large surface area (~2630 m^2^·g^−1^) [17,18,19]. Graphene has shown dramictical enhancement in electrical, mechanical, thermal, and barrier properties of polymer composites at low concentrations [18,19,20,21]. Usually, there are three methods for fabriction of graphene/polymer composites: solution mixing, melt compounding, and in situ polymerization [19,22]. Compared to the former two methods, the in situ polymerization not only can make graphene sheets well-dispersed in polymer matrix, but also enable chemical bonds formed between graphene and polymer readily. Therefore, a variety of graphene-based polymeric composites, such as graphene/polystyrene nanocomposites [23,24], graphene/epoxy nanocomposites [25,26,27] and graphene/UPR nanocomposites [28,29,30] have been prepared through this method.

In our previous work, we prepared tung oil (TO)-modified UPRs via intermolecular Diels–Alder (D–A) addition between unsaturated polyesters and TO triglycerides [31]. The obtained biobased UPRs also demonsatrated a large drop in stiffness when the TO content was larger than 7.4 wt % of UPR (with styrene). Thus, in this study, graphene was empolyed to reinforce a TO-based UPR. To the best of our konwledge, graphene has never been used to reinforce biobased UPRs. Using graphene oxide (GO) as starting material, we prepared the graphene-reinforced TO-based UPRs by in situ polymerization combined with Diels–Alder (D–A) addition. Our goal is to recovery the loss of stiffness for tung oil-based UPRs, and to see whether balanced stiffness–toughness can be achieved.

## 2. Experimentals

### 2.1. Materials

Tung oil was purchased from Jiangsu Donghu oil Co., Ltd. (Yancheng, China), which has a specific gravity of 0.935−0.940 at 25 °C. Graphene Oxide was obtained from Nanjing XFNANO Materials Tech Co., Ltd. (Nanjing, China), which has a lateral size of 0.5−5 μm and thickness of 0.8−1.2 nm. Maleic anhydride (MA), phthalic anhydride (PA), styrene (≥99%), and hydroquinone were obtained from Sinopharm Chemical Reagent Co., Ltd. (Shanghai, China) Ethanol (≥99%), propylene glycol (PG) (99%), and toluene (≥99.5%) were obtained from Nanjing Chemical Reagent Co., Ltd. (China) (≥99%) Dibutyl phthalate (≥99.5%) and *N*,*N*-dimethylaniline (≥99%) were obtained from Shanghai Lingfeng Chemical Reagent Co., Ltd. (Shanghai, China) Benzoyl peroxide (≥99%) was obtained from Shanghai Macklin Biochemical Co., Ltd. (Shanghai, China) The PG and styrene were dried by molecular sieves for at least one week before use.

### 2.2. Synthesis of Neat UPR

50.22 g of PG, 39.22 g of MA, 29.62 g of PA, and 0.119 g of hydroquinone were added into a 250 ml four-necked flask equipped with a mechanical stirrer, a thermometer, a N_2_ gas inlet, and a fractionating device. The mixture was then heated to 60 °C, agitated at 60 °C for 0.5 h, heated to 160 °C under N_2_ protection, and maintained at 160 °C for 1.5 h. Subsequently, the mixture was heated to 200 °C and reacted at 200 °C until the acid value of the system decreased to a set value (around 33 mgKOH/g). After that the reaction temperature was reduced to 120 °C and 0.1 g of hydroquinone was added and mixed for 0.5 h. Finally, the temperature was lowered to 90 °C and 58.30 g of styrene (about 35% of the total resin weight) was added and blended with the resultant mixture for 1 h. A colorless and transparent liquid resin was produced.

### 2.3. Synthesis of UPR/TO

The UPR/TO resin was synthesized via melt polycondensation incorporated with D–A addition. The procedure can be divided into two stages. The first stage involved the synthesis of unsaturated polyester, as described in the above section. The acid value also reached the set value. At the second stage, the reaction temperature was lowered to 120 °C, 0.1 g of hydroquinone was added, and 16.65 g of TO was added dropwise into the flask within 0.5 h. At last, 58.30 g of styrene was added into the mixture and mixed at 90 °C for 1 h. A light yellow and translucent liquid resin was obtained.

### 2.4. Preparation of UPR/TO/GO Composites

**Figure 1:**
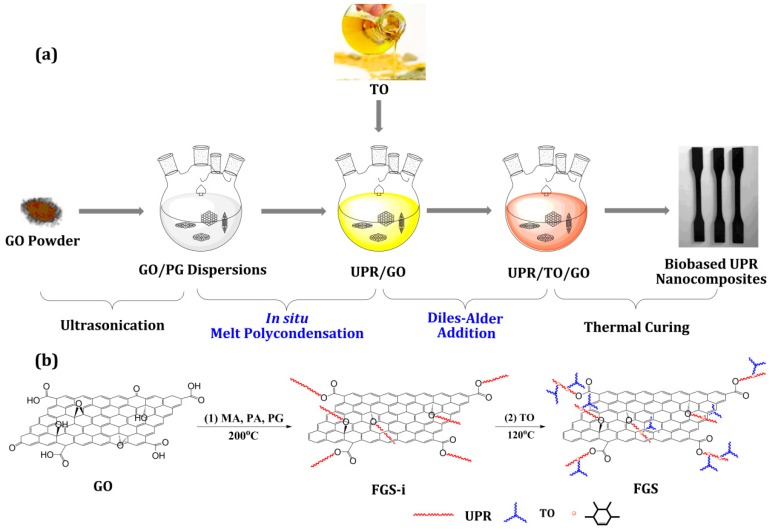
(**a**) The prepatration of graphene-reinforeced biobased unsaturated polyester nanocomposites via a combination of in situ melt polycondensation and Diels–Alder addition; (**b**) possible chemical changes of graphene oxide (GO) during the in situ preparation of biobased nanocomposites.

The synthesis of UPR/TO/GO nanocomposites was carried out in three basic stages, as shown in Figure 1. In the first stage, 50.22 g of PG and an appropriate amount of GO powder were put into a 250 mL four-neck flask and ultrasonicated for 2 h to achieve a homogeneous GO/PG dispersion. In the second stage, a reaction mixture of MA (39.22 g), PA (29.62 g), and hydroquinone (0.119 g) was added into the 250 mL flask and the reaction was conducted identically to the procedures indicated in the first stage of synthesizing UPR/TO. The third stage was exactly the same as the second stage of UPR/TO. At the end, a black and opaque liquid resin was attained. In our experiments, the content of TO in the obtained UPR/TO and UPR/TO/GO composites was always 10 wt % of the UPR resin (including styrene). The content of GO in the UPR/TO/GO composites was 0.05, 0.10, 0.15, 0.20 and 0.30 wt % of the UPR resin (including styrene), thus for simplicity the corresponding nanocomposites were denoted as UPR/TO/G0.05, UPR/TO/G0.10, UPR/TO/G0.15, UPR/TO/G0.20, and UPR/TO/G0.30, respectively.

During the manufacturing process, GO could be simultaneously grafted by unsaturated polyesters and thermally reduced due to the high temperature involved in the melt polycondeansation, and the grafted unsaturated polyesters could further graft with TO via D–A addition, thus new functionalized graphene sheets (FGS) would be produced (Figure 1b). Successive centrifugation/redissolution cycles were performed to separate FGS from the obtained polymer composites. Typically, 30 g of the UPR/TO/G0.15 polymer composite (without styrene) was dissolved in 150 mL of toluene/ethanol mixed solvent (50/50, *v*/*v*), stirred for 2 h to remove the absorbed polymers from the graphene surface, and then centrifuged at 11,000 rpm for 30 min to precipitate the graphene completely. The attained centrifugate was dissolved by toluene/ethanol solvent and separated by centrifugation repeatedly for 5 times. At last, the obtained centrifugate was washed with ethanol twice, then dried at 50 °C for 4 h and under vacuum for another 48 h. The resulting black solid material was labeled as FGS. In an effort to further determine the contents of graphene, UPR, and TO in the FGS, a referenced polymeric nanocomposite were prepared according to the fabricating procedure of UPR/TO/G0.15 but without adding TO. The obtained composite was named as UPR/G0.15. By the same repeating centrifugal-washing procedure, a similar black solid material was obtained, which was labeled as FGS-i since it was an intermediate compared to the FGS.

### 2.5. Curing of UPR, UPR/TO, and UPR/TO/GO Composites

The as-fabricated UPR, UPR/TO, and UPR/TO/GO composites were all cured in a same procedure. Typically, the resin samples were blended with the initiator (2 wt % of the total resin) for 30 min and with the promoter (0.2 wt % of the total resin) for 3 min, degassed, poured rapidly into homemade polytetrafluoroethylene molds, cured at room temperature for 3 h, and postcured at 80 °C for 12 h.

### 2.6. Characterization

Acid values were determined based on the procedures presented in GB/T 2895–1982. Atomic force microscopy (AFM) images were obtained using a SPM-9600 atomic force microscope (Shimadzu, Kyoto, Japan) in taping mode. FT-IR spectra were obtained using a Nicolet iS10 IR spectrometer (Thermo Fisher, Waltham, MA, USA). Thermogravimetric analysis (TGA) was conducted on a STA 409PC thermogravimetry instrument (Netzsch, Selb, Germany). X-ray photoelectron spectroscopy (XPS) was performed on a AXIS Ultra^DLD^ photoelectron spectrometer (Shimadzu, Kyoto, Japan). Raman spectra were collected on a DXR532 Raman spectrometer with a wavelength of 532 nm (Thermo Fisher, Waltham, MA, USA). Scanning electron microscopy (SEM) examinations were studied on an S-3400N scanning electron microscope (Hitachi, Tokyo, Japan). Transmission electron microscopy (TEM) examinations were carried out by a Tecnai G^2^20 transmission electron microscope (FEI, Hillsboro, OR, USA). Tensile and flexural tests were conducted on a SANS7 CMT-4304 universal tester (Xinsansi, Shenzhen, China). Impact tests were conducted on a CEAST 9050 impact tester (Instron, Norwood, MA, USA). All the mechanical tests followed the procedures presented in the GB/T 2567-2008. Dynamic mechanical analysis (DMA) was performed on a Q800 solids analyzer (TA, New Castle, DE, USA) in three-point bending mode.

## 3. Results and Discussion

### 3.1. Structural Characterization of FGS

For polymer nanocomposites, it is well known that if the nanofiller and polymer matrix form chemical bonds, the interfacial interactions between the polymer and nanofiller will be improved greatly. To confirm whether chemical bonds are generated between the nanofiller and polymer, the chemical state of graphene nanosheets should be determined first. Thus FGS was extracted from the biobased UPR/TO/G0.15 composite by the repeating centrifugal-washing method and characterized by sedimentation experiments, AFM, FT-IR, TGA, XPS, and Raman spectroscopy, as discussed below. For comparison, GO and thermally reduced GO (RGO) (prepared by treating neat GO powder at 200 °C for 10 h, similar condition to the in situ polycondensation) were also characterized.

Firstly, sedimental experiments were taken to assess their solubility in organic solvent. GO, RGO, and FGS were dispersed in ethanol by stirring for 5 min and probe-sonicating for 5 min, and then precipitated for 30 min.

**Figure 2:**
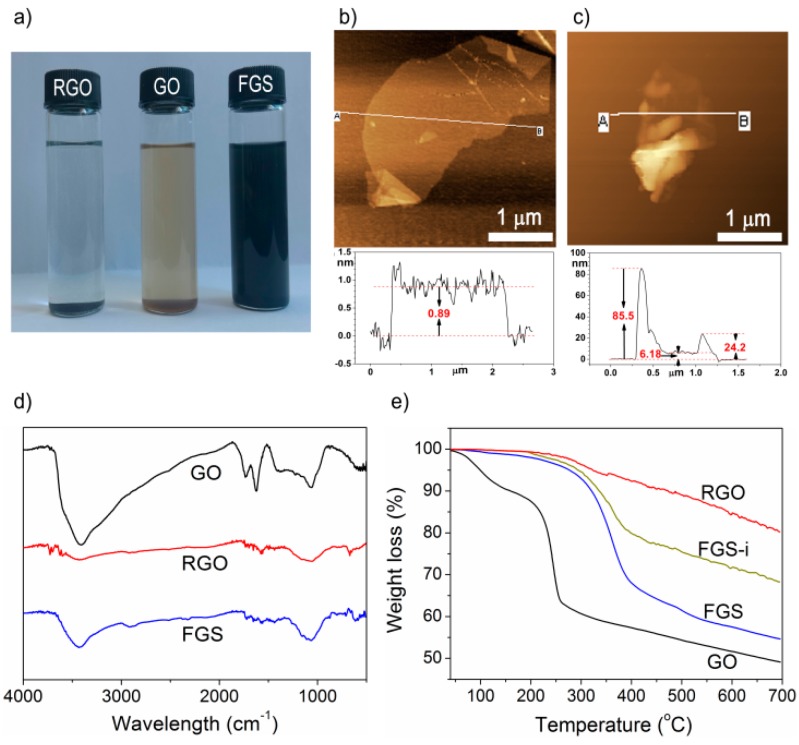
(**a**) Sedimentation experiment; (**b,c**) atomic force microscopy (AFM) images of GO and FGS; (**d**) FT-IR spectra of GO, thermally reduced GO (RGO), and FGS; (**e**) TGA curves of GO, RGO, FGS-i, and FGS.

The resulting photographs are displayed in Figure 2a. Both FGS and RGO were black from appearance, while GO was golden yellow, indicating GO was reduced during the polycondensation. In addition, GO and RGO precipitated apparently, whereas FGS had no obvious preciptation. This difference indicated that FGS possessed better solubility than both GO and RGO, which may be attributed to that FGS were grafted by organic polymers. AFM was employed to further characterize the graphene samples. The typical tapping-mode AFM images of GO and FGS are presented in Figure 2b,c, respectively. The thickness of GO nanosheet was about 0.89 nm, which was in good accordance with the results from literature [28,32,33]. In contrast, the FGS possessed dinstinct thicknesses: at the thinest point it was only 6.18 nm, while at some other places it could reach dozens of nanometers (eg. 24.2 nm). The results suggested that graphene sheets were not only grafted by linear polymers like unsautrated polyesters, but could also be partially grafted by crosslinked polymers, since the grafted unsaturated polyesters could form crosslinking polymers with TO triglycerides [31]. The crosslinked polymers could be directly observed in the lower half of FGS’s image (Figure 2c), where the nanosheet were rolled up in some extent due to the crosslinking effect of TO with the grafted polyesters. Figure 2d provides the FT-IR spectra of GO, RGO, and FGS. It can be seen that the spectrum of FGS was analegous to RGO but quite different from that of GO. The original bands in GO, such as carboxyls (3400–2500 cm^−1^) and carbonyl (~1730 cm^−1^), decreased obviously in intensity for FGS sample, indicating the reduction of GO to FGS. At last, samples of GO, RGO, FGS, and FGS-i were analyzed by TGA (Figure 2e). In the curve of GO there was two main weigh loss stages at around 100 and 250 °C, which are attributed to the removal of absorbed water and the pyrolysis of oxygen-containing functional groups, respectively [32,33]. The total weight loss below 250 °C was approximate 40%. In the curve of RGO, the degradation rate was slow, suggesting RGO was more stable than GO. In contrast, both the TGA curves of FGS-i and FGS exhibited only one main stage of weight loss at 270–450 °C, which is ascribed to the degradation of the grafted polyesters or biopolyesters. No apparent weight loss stages were found below 250 °C, suggesting that the original oxygenated groups on the surface were removed or grafted by polymers. From the main weight loss stage of FGS-i, the weight fraction of grafted unsaturated polyesters on the graphene surface could be estimated and was about 25%; while from the same stage of FGS, the total weight fraction of grafted polyesters and TO was about 35%. Thus it can be deduced that the FGS contained around 65% of graphene, 25% of unsaturated polyesters, and 10% of TO molecules.

**Figure 3:**
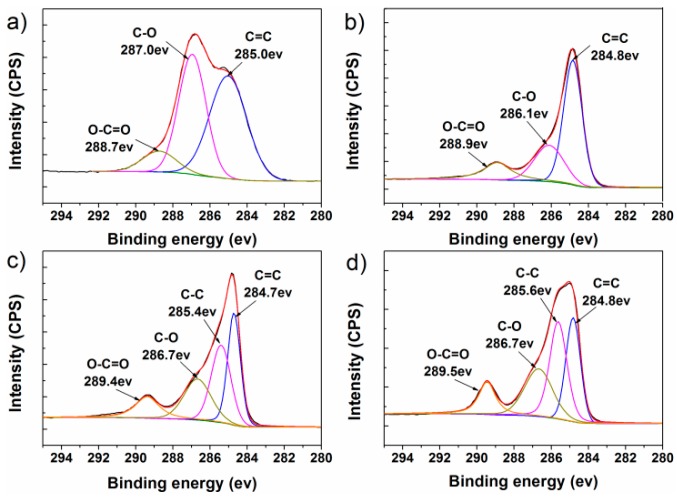
The C 1s peaks in XPS spectra of (**a**) GO, (**b**) RGO, (**c**) FGS-i, and (**d**) FGS.

XPS was conducted to further analyze the graphene-based compounds, as shown in Figure 3a–d. In the spectra of GO, only three peaks were depicted: sp^2^ C=C (~285.0 eV), C–O (~287.0 eV), and O–C=O (~288.7 eV). The RGO spectra also comprised the three peaks, but the intensities of oxygen-containing peaks at 286.1 and 288.9 eV (relative to the C=C peak) decreased obviously. In both the spectra of FGS-i and FGS, the three peaks had a similar trend as those in RGO, indicating the reduction of GO. Differently, new peaks occurred at 285.4 or 285.6 eV, which means the ocurrence of sp^3^ C–C bonds [34,35,36,37]. The bonds probably came from the grafted polyesters and TO molecules which involved many sp^3^ C–C bonds. Compared to FGS-i, the intensity of new C–C peak in FGS spectra increased (relative to that of C=C peak) due to the incorporation of TO. The C/O atomic ratios for all the graphene nanomaterials, neat UPR, and TO were caculated and the related data are listed in Table 1. With the C/O ratios of UPR, RGO, and FGS-i, the weight content of grafted unsaturated polyesters onto graphene can be estimated and was about 28%; with the ratios of TO, FGS-i, and FGS, the grafted TO can also be determined and was around 5%. These values are close to the calculated values from the TGA results.

**Figure 4:**
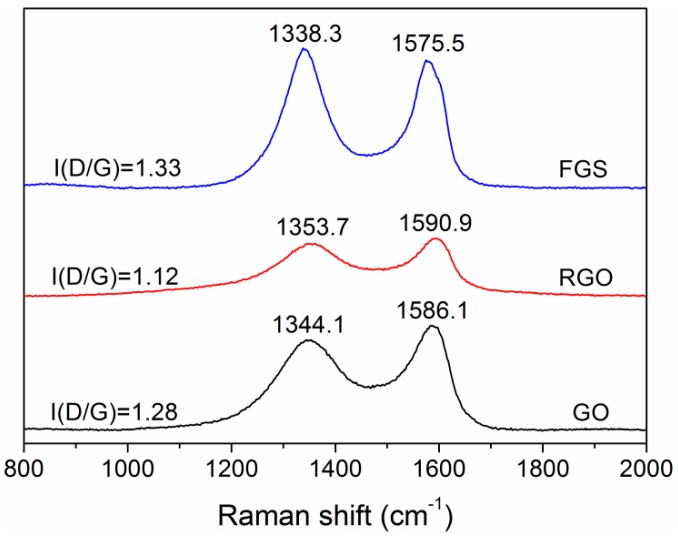
Raman spectra of GO, RGO, and FGS.

Raman spectroscopy has been reported as a powerful probe for the structures of carbonaceous materials. Figure 4 gives the Raman spectra of GO, RGO, and FGS. The GO spectrum displayed a D band at 1344.1 cm^−1^ and G band at 1586.1 cm^−1^ which are ascribed to the breathing mode of *κ*-point phonons of A_1g_ symmetry and the first-order scattering of the the E_2g_ phonons, respectively [33,34]. In the spectrum of RGO, the G band blue-shifted to 1590.9 cm^−1^, which was close to the value of raw graphite. In the spectrum of FGS, the D and G bands shifted to 1338.3 and 1575.5 cm^−1^. The red-shift of G band can be attributed to that the defects of FGS were more apparent than those of GO. The intensity ratio of D and G bands (I(D/G)), corresponding to the disordered and ordered crystal structures of carbon, is inverse to the average size of sp^2^ domains [33,34,36]. The I(D/G) value decreased from 1.28 for GO to 1.12 for RGO, which was in good agreement with the fact that RGO was reduced GO. In contrast, the value of FGS (1.33) was a little higher than that of GO. The reason for the slight increase may lie in that the grafted polymers make the defects of graphene more distinct than the orginal ones.

In conclusion, the original GO nanomaterial was grafted by unsaturated polyesters and simultanesously reduced during in situ polymerization, and followed by grafting TO triglycerides onto polyesters via D–A addition. Therefore, it can be inferred that the interfacial interactions between the polymer matrix and graphene nanosheets were strong.

### 3.2. Dispersions of Graphene in Polymer Matrix

**Figure 5:**
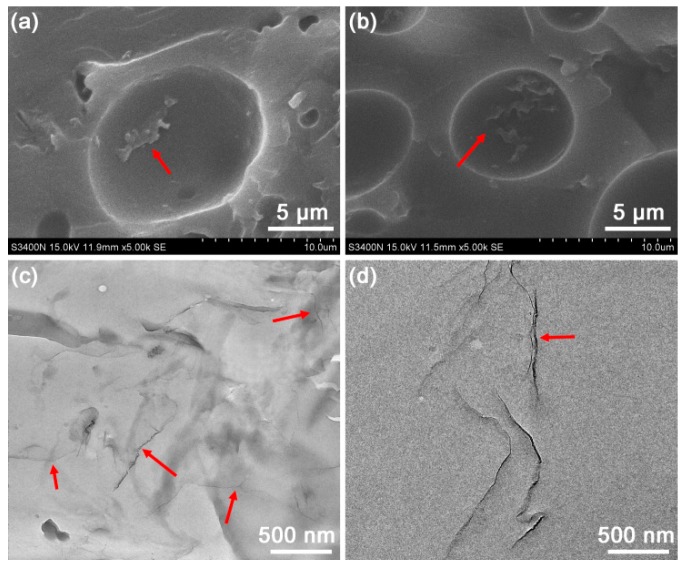
SEM images of the etched UPR/TO/GO composites with GO contents of (**a**) 0.10% and (**b**) 0.30%; TEM images of the UPR/TO/GO composites with GO contents of (**c**) 0.10% and (**d**) 0.30%.

Apart from the interaction between the nanofiller and polymer, disperison state of the nanofiller in the polymer matrix is another key factor that influences the ultimate properties of fabricated composites. SEM and TEM techniques can provide the direct observation of dispersion behaviours of nanofillers. Figure 5a,b shows the SEM images of tensile fracture surfaces of the biobased nanocomposites with GO contents of 0.10% and 0.30%, respectively. Before the SEM observation the fracture surface of tensile-testing specimen were etched in a 10% NaOH/ethanol solution at 40 °C for 2 h. The FGS was evenly dispersed in the UPR/TO/G0.10 composite, whereas the graphene sheets were much closer to each other in the UPR/TO/G0.30 composite. Moreover, the protruding graphene sheets in both the nanocomposites were still coated with adsobed resins after etching, reflecting the strong filler–polymer interfacial interaction [28,38]. It should be mentioned that phase separation like craters occurred in both the polymer matrices, which is attibuted to the excess of TO [31,39]. Figure 5c,d presents the TEM images of the UPR/TO/G0.10 and UPR/TO/G0.30 samples. The graphene nanosheets were exfoliated much better and dispersed more homogeneously in the UPR/TO/G0.10 composite (as indicated by the red arrows) than in the UPR/TO/G0.30 composite, which was consistent with the SEM results. The nanosheets in UPR/TO/G0.30 demonstrated some extent of aggregation. All these morphologies would affect ulitmate properties of the cured composites.

### 3.3. Properties of the UPR/TO/GO Biobased Nanocomposites

In this work, the most noteworthy questions are whether the stiffness of the biobased UPRs can be completely recovered by the incorporation of graphene and whether the comprehensive properties of the resulting composites can compare to those of petroleum-based UPR. Thus mechanical and thermal properties of the obtained biobased UPR nanocomposites were investigated carefully.

**Figure 6:**
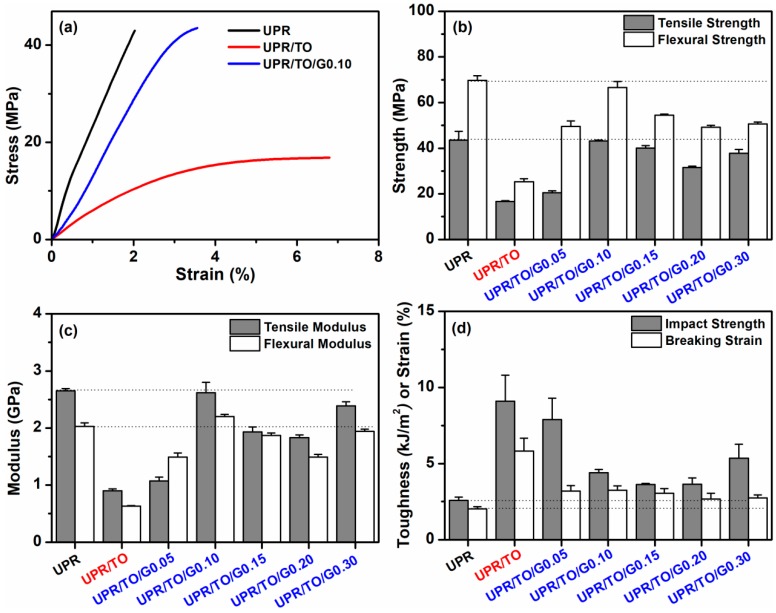
(**a**) Stress–strain curves of neat UPR, UPR/TO, and UPR/TO/G0.10; (**b**) tensile and flexural strengths, (**c**) tensile and flexural moduli, and (**d**) impact strength and tensile breaking strain of UPR, UPR/TO, and UPR/TO/GO composites.

Figure 6a illustrates the typical stress–strain curves of neat UPR, UPR/TO, and UPR/TO/G0.10. The UPR/TO/G0.10 composite showed a comparable stress to that of neat UPR, indicating that the prepared composite could be as good as the neat UPR in stiffness. Meanwhile the strain of UPR/TO/G0.10 was larger than that of UPR, suggesting the toughness of UPR/TO/G0.10 was even better. In addition, compared to the UPR/TO, although the incorporation of GO resulted in the decrease of strain, the stress of UPR/TO was improved remarkably. The improvement can be attributed to the good exfoliation of FGS and the strong interaction between FGS and polymer matrix, which can efficiently transfer the load from the matrix to the graphene nanofiller [19,22,28,32,33,40].

Figure 6b presents tensile and flexural strengths of neat UPR, UPR/TO, and UPR/TO/GO composites. The tensile and flexural strengths of neat UPR were 43.5 and 69.7 MPa, respectively. By the addition of 10% TO, the values decreased to 16.7 and 25.3 MPa, respectively, which results from a second phase generated by unreacted tung oil [31,39], as shown in Figure 5a,b. As the GO content increased, both the strengths increased firstly and decreased later. The increase is caused by the enhancement effect of the FGS filler, while the decrease probably results from the poor dispersion of graphene like aggregation. The composites possessed an optimal loading level at 0.10% of GO. At this concentration the tensile and flexural strengths reached 43.2 MPa and 66.6 MPa, which was only 0.59% and 4.45% inferior to those of neat UPR, respectively. Nevertheless, they increased by 159% and 163% compared to UPR/TO, respectively, which can be ascribed to the sufficient exfoliation of graphene nanosheets and the strong interaction of FGS–polymer too.

Figure 6c demonstrates tensile and flexural moduli of neat UPR, UPR/TO, and UPR/TO/GO composites. These properties can represent the stiffness of polymer materials. The tensile and flexural moduli of the neat UPR were 2.65 and 2.03 GPa, respectively. By the incorporation of 10% TO, these values decreased to 0.90 and 0.63 GPa, respectively, which is due to the phase separation too. For the UPR/TO/GO composites, the moduli showed a similar trend as the strengths when the GO concentration grew. The composite at the GO content of 0.10% showed a tensile modulus of 2.62 GPa, which was 1.13% lower than that of UPR. Yet the flexural modulus of the composite reached 2.20 GPa, which was 8.37% higher than that of neat UPR. Therefore, the stiffness of the UPR/TO/G0.10 composite could be regarded as equivalent to that of neat UPR. Notably, the two values increased by 191% and 250% compared to those of UPR/TO, respectively, which can be also attributed to the complete exfoliation of FGS and the strong interfacial interaction between graphene and polymer matrix.

Figure 6d provides impact strength and tensile breaking strain of neat UPR, UPR/TO, and UPR/TO/GO composites. The two values of neat UPR were 2.58 kJ/m^2^ and 2.03%, respectively. After the addition of 10% TO, they increased rapidly to 9.09 kJ/m^2^ and 5.82%, respectively, which is caused by the effect of phase separation [8,9,31]. As the content of GO increased, both the data decreased gradually. As is known to us, the addition of nanofillers leads to a decrease of toughness [16,25,41]. The two values for the UPR/TO/G0.10 composite were 4.40 kJ/m^2^ and 3.24%, which were 70.5% and 50.2% higher than those of pure UPR, respectively. These results suggested that the toughness and flexibility of UPR/TO/G0.10 were obviously better than those of neat UPR.

**Figure 7:**
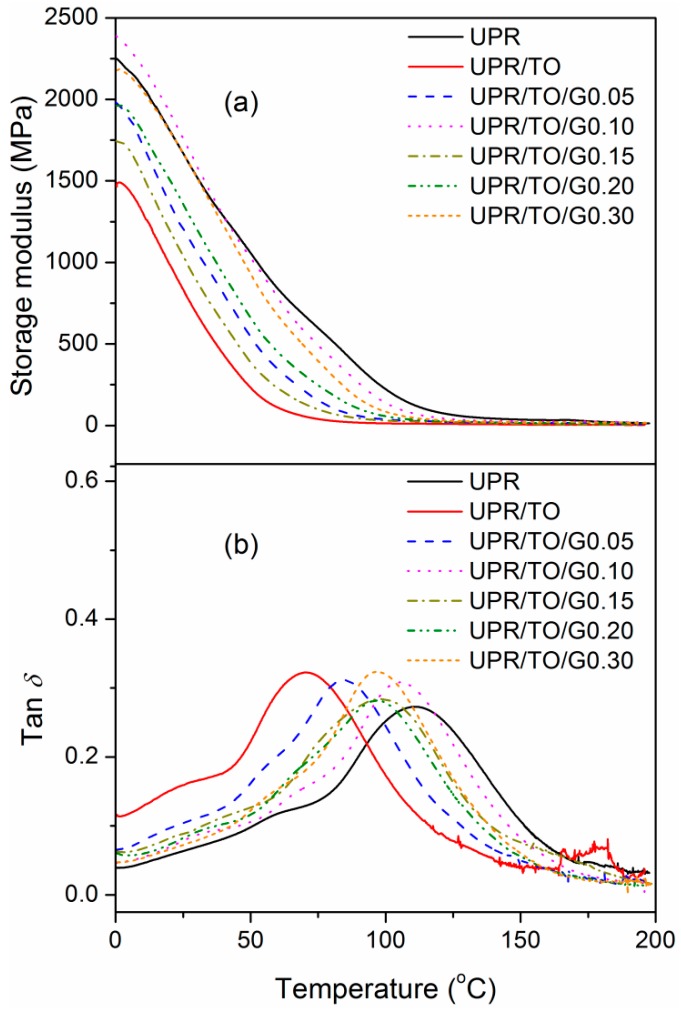
(**a**) Storage modulus and (**b**) Loss factor of neat UPR, UPR/TO, and UPR/TO/GO composites.

DMA was performed to study the thermo-mechanical properties of neat UPR, UPR/TO, and UPR/TO/GO composites, as shown in Figure 7. The related results are presented in Table 2. The storage modulus at 25 °C (*E*′_25_), which can also reflect the stiffness, was 1.67 GPa for neat UPR. By the incorporation of 10% TO, the data decreased to 0.83 GPa due to the phase separation. As the increase of GO content, the value also experienced a firstly-increasing and subsequently-decreasing process, and showed a rebound at 0.30% of GO. The optimal value occurred at 0.10% of GO and was 1.76 GPa, which was 5.39% and 112% higher than those of UPR and UPR/TO, respectively. Glass transition temperature (*T*_g_) can be determined from the peaks of loss factors (tan *δ*). The variation of *T*_g_ demonstrated an analogous trend as *E′*_25_. The *T*_g_ of UPR/TO/G0.10 was 105.2 °C, which was only 4.88% lower than neat UPR’s (110.6°C) but 49.4% higher than that of UPR/TO (70.4 °C). The improvements of *E′*_25_ and *T*_g_ for UPR/TO/G0.10 are ascribed to the good exfoliation of FGS and the strong FGS–polymer interaction too.

**Figure 8:**
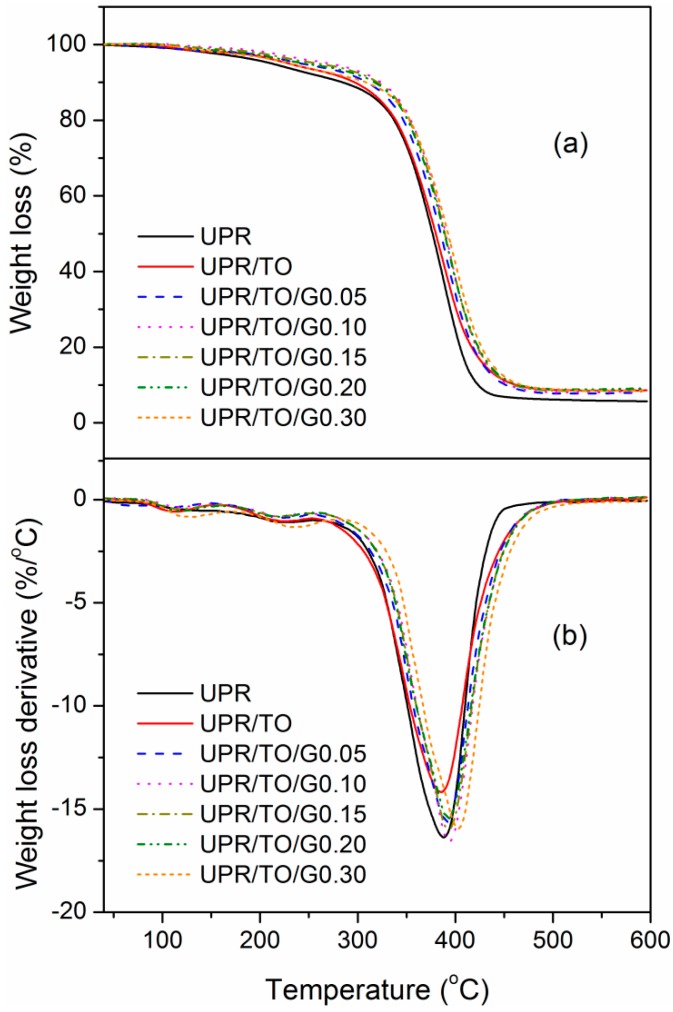
(**a**) TGA curves and (**b**) their derivatives of neat UPR, UPR/TO, and UPR/TO/GO composites.

TGA was carried out to investigate thermal stability of neat UPR, UPR/TO, and UPR/TO/GO composites, as indicated in Figure 8. The thermal-property data, including 5% weight-loss temperature (*T*_5_), peak temperature at the curve of weight–loss rate (*T*_p_), and char yield (*w*_char_) are summarized in Table 2. All the properties possessed similar trends of variation as *E′*_25_ or *T*_g_ when the GO content increased. For the neat UPR they were 212.2 °C, 388.1 °C, and 5.70%, respectively. And for the UPR/TO/G0.10 composite they were 270.6 °C, 392.9°C, and 8.37%, which were 27.5%, 1.24%, and 46.8% larger than those of neat UPR, respectively. Therefore, the thermal stability of UPR/TO/G0.10 composite was apparently superior to that of neat UPR.

## 4. Conclusions

We sucessfully prepared the novel graphene-reinforced biobased UPR nanocomposites via in situ melt polycondensation intergrated with D–A addition. The fabricating method can strengthen the interfical interactions between the graphene, polymer, and biomodifier and does not involve any solvent, which is efficient and ecofriendly. Notably, the stiffness of TO-based UPR was greatly enhanced by only incoporating 0.10% of GO, and the comprehensive properties of the resulting biobased UPR nanocomposite were even superior to the petroleum-based UPR. The excellent performance can be attributed to the well exfoliation of FGS and the strong interactions between FGS and polymeric matrix. Consequently, the developed UPR/TO/GO composites show great potential in the UPR industry and the fabricating method can be employed for other biobased polyester nanocomposites.

## Figures and Tables

**Table 1:** XPS data for all the graphene nanomaterials, neat UPR, and TO.

Sample	Relative Atomic Percentage (%)		C/O Atomic Ratios
	C	O	
GO	69.0	31.0	2.23
RGO	81.9	18.1	4.53
FGS-i	78.5	21.5	3.72
FGS	80.1	19.9	4.02
UPR	62.5	37.5	1.67
TO	90.5	9.5	9.5

**Table 2:** Results of DMA and TGA for neat UPR, UPR/TO, and UPR/TO/GO composites.

Sample	*E′*_25_^a^ (GPa)	*T*_g_^b^ (°C)	*T*_5_^c^ (°C)	*T*_p_^d^ (°C)	*w*_char_^e^ (%)
UPR	1.67	110.6	212.2	388.1	5.70
UPR/TO	0.83	70.4	229.6	385.5	8.61
UPR/TO/G0.05	1.21	84.3	241.7	391.3	8.08
UPR/TO/G0.10	1.76	105.2	270.6	392.9	8.37
UPR/TO/G0.15	1.04	98.4	259.4	392.9	8.76
UPR/TO/G0.20	1.36	96.4	246.1	392.9	9.16
UPR/TO/G0.30	1.67	97.1	229.1	397.8	8.55

*^a^* Storage modulus at 25 °C. *^b^* Glass transition temperature. *^c^* 5% Weight–loss temperature. *^d^* Peak temperature at the curves of weight-loss rate. *^e^* Char yield.
